# Fermentative profile, losses and chemical composition of silage soybean genotypes amended with sugarcane levels

**DOI:** 10.1038/s41598-020-78217-1

**Published:** 2020-12-03

**Authors:** Anderson de Moura Zanine, Orgélio Augusto de Sene, Daniele de Jesus Ferreira, Henrique Nunes Parente, Michelle de Oliveira Maia Parente, Ricardo Martins Araújo Pinho, Edson Mauro Santos, Thiago Vinicius Costa Nascimento, Anny Graycy Vasconcelos de Oliveira Lima, Alexandre Fernandes Perazzo, Ygor Nascimento Portela, Danrley Martins Bandeira

**Affiliations:** 1 Department of Animal Science, Federal University of Maranhão, MA-230, KM 04, s/nº-Boa Vista, Chapadinha, Maranhão 65500-000 Brazil; 2 Crato Campuses, Federal Institute of Education, Science and Technology of Ceará, Crato, Ceará Brazil; 3 Department of Animal Science, Federal University of Paraíba, Areia, Paraíba Brazil

**Keywords:** Bacteria, Fungi, Natural variation in plants, Plant development, Plant physiology, Microbiology

## Abstract

The experiment aimed to evaluate the fermentative and nutritional profile of the silage of four soybean plant genotypes (BRS 333 RR, Pampeanas: C50, C60, and C70) ensiled with levels of sugarcane (0, 25, 50, 75, and 100%). The experiments were conducted in a completely randomized design, in factorial scheme 4 × 5 (four soybean genotypes and five levels of sugarcane inclusion) with four replicates. Silages with 100% soybean plant presented the highest levels of butyric acid (P < 0.001) and ammoniacal nitrogen (P < 0.047); however, the intermediate addition of sugarcane contributed to lactic fermentation (P < 0.001). Besides, there was a quadratic effect (P < 0.05) for the recovery of dry matter, which ranged from 83.28 to 95.29%, with higher values observed for silage with the same proportions of soybean plant and sugarcane. It was verified that the crude protein content exhibited decreasing linear effects (P < 0.001), varying among 4.60 to 7.48% in the silages. It was concluded that the highest recovery of dry matter, the best fermentation profile, and the highest levels of crude protein and digestibility occurred in the inclusion between 25 and 50% of sugarcane in soybean silage, with the superiority of the C50 soybean genotype.

## Introduction

The absence of rain for a prolonged period of time is the main problem in the agricultural sector with the potential to disable large areas of agricultural exploitation in the world^1–3^. Development and improvement of technologies to food production and evaluation could support production systems, mitigating productive, economic, and climatic adversities. Brazil is one of the largest soybean producers in the world. In the 2019 harvest, national production reached 123.5 million tons, in a planted area of 35 million hectares^4^.

Soybean plant is grown worldwide at latitudes greater than 30°, where temperate climate conditions prevail, however Brazil is an exception by development hybridized soybean genotypes that are adapted to produce in regions of tropical and subtropical climates. These genotypes were selected to have a long youthful period, i.e. flowering is later, making vegetative growth greater by increasing biomass production and decreasing the proportion of grains in relation to leaves and stem, besides lengthening the soybean crop cycle, that varies from 100 to 140 days, divided into vegetative and reproductive stages^5–8^. The reproductive stage is where the formation of pods and soybean grains occurs, from stages R 5 and 6, after these stages the soybean plant tends to decrease the chemical composition of the plant for the formation and maturation of soybean grains^6,7^.

Several factors may influence grain production, for instance, climatic variability, which sometimes affects the water availability for the crop, even in the rainy season^8–10^. Also, these factors can affect some morphological characteristics of plants, such as height, biomass production, and seed production^10,11^, in these cases, it limits the seed viability, but not for the production of silages. Regarding grain productivity, the occurrence of mutagenic soybean can affect up to 60%. However, despite being affected, these plants produce biomass^11^, which can also be used to be ensiled. In this sense, considering the relevance of this plant in the world, studies of conservation in the form of silage is an alternative for the production of ruminant feed.

Soybean, as well as other legumes, have a high amount of crude protein (CP), low content of soluble carbohydrates, and high buffering power^9,11,12^. The interaction of these factors in the silo can decrease the speed of reducing the pH value and, thus, result in inadequate lactic fermentation^9,12,13^, which makes its silage a challenge, can be researched the use of some additives or mixed silage with grass species, to compensate for the aforementioned characteristics that hinder the proper fermentation of biomass.

An alternative that may be viable is the use of sugarcane that has a high content of water-soluble carbohydrates may be the supplement of the energy demand of lactic acid bacteria (LAB) in the fermentation process of silage^14^. However, a large amount of soluble carbohydrates and epiphytic yeasts from sugarcane may lead to ethanol production, CO_2_ and water, and, consequently, lead to dry matter losses^15^. Besides, sugarcane forage has low levels of CP and minerals, in addition to high levels of fiber^13–15^.

Thus, mixed silage with two or more species or genotypes, such as grasses and legumes, should be a significant agricultural practice in complementary situations. Despite being crops with usually different harvest times, if harvested together at the end of the rainy season they can add positive characteristics from both crops^15,16^. In other words, factors that hinder the silage process of the isolated soybean and sugarcane plant could be canceled with the appropriate combination in the silage of these forage plants since the favorable and unfavorable characteristics of both would be strategically improved.

In this sense, the present study aimed to enhance the fermentative and nutritional ensilage profile of whole plants of different soybean genotypes through the addition of soluble carbohydrates from the inclusion of sugarcane.

### Results

There was an interaction (P < 0.001) between the levels of sugarcane and silages of soybean genotypes for the contents: lactic acid; acetic acid; ratio of lactic acids and acetic acid; propionic acid; butyric acid; ethanol; and ammoniacal nitrogen (N-NH_3_/NT) (Table 1).

**Table 1:** Probability values for inclusion levels of sugarcane, soybean genotypes, and the interaction of the contents of organic acids, ethanol, and ammoniacal nitrogen in silages.

^1^VS	Organic acids					Ethanol	^7^ N-NH_3_
	^2^LA	^3^AA	^4^LA:AA	^5^AP	^6^AB		
Levels	< 0.001	< 0.001	0.017	< 0.001	< 0.001	< 0.001	< 0.001
Genotypes	< 0.001	< 0.001	< 0.001	< 0.001	< 0.001	< 0.001	0.047
^8^ N x ^9^G	< 0.001	< 0.001	< 0.001	< 0.001	< 0.001	< 0.001	< 0.001

^1^Variation source; ^2^lactic acid; ^3^acetic acid; ^4^relation between lactic and acetic acids; ^5^propionic acid; ^6^butyric acid; ^7^ammoniacal nitrogen, ^8^levels of sugarcane; ^9^soybean genotypes.

It was observed a positive quadratic effect (P < 0.001) of the inclusion of sugarcane in the silage from all soybean genotypes for lactic acid contents with maximum points of 17.03, 18.79, 14.71, and 14.16 at the sugarcane inclusion levels of 50%, to soybean genotypes BRS, C60, C50, and 75% to C70 soybean genotype (Table 2). Moreover, silages with C70 soybean genotypes showed higher means of lactic acid (P < 0.05).

**Table 2:** Mean values of the contents of organic acids and ammoniacal nitrogen in silages of soybean genotypes with addition levels of sugarcane.

Soybean genotypes	Inclusion levels in sugarcane					SEM		P value		Mean	Equation
	0	25	50	75	100			L	Q		
**Lactic acid (LA) %**											
BRS	3.71	11.79	17.03	9.38	4.48	1.132		0.367	< 0.001	9.28B	Y = 3.5572 + 0.4474x − 0.0044x^2^
C70	0.02	18.10	16.20	18.79	4.48	1.789		< 0.001	< 0.001	11.52A	Y = 4.8322 + 0.6504x − 0.0069x^2^
C60	0.41	14.36	14.71	10.85	4.48	1.296		< 0.001	< 0.001	8.96C	Y = 3.4853 + 0.4938x − 0.0051x^2^
C50	0.73	12.57	14.16	8.20	4.48	1.147		0.001	< 0.001	8.03D	Y = 3.1354 + 0.4292x − 0.0044x^2^
**Acetic acid (AA) %**											
BRS	1.47	1.07	1.71	1.50	3.79	0.229		< 0.001	< 0.001	1.91B	Y = 3.5572 + 0.4474x − 0.0044x^2^
C70	2.97	1.58	1.60	1.34	3.79	0.230		0.020	< 0.001	2.26B	Y = 3.5903 − 0.0901x + 0.0008x^2^
C60	3.67	1.36	2.16	4.65	3.79	0.354		0.067	0.047	3.13A	Y = 4.4863 − 0.0665x + 0.0005x^2^
C50	2.55	0.82	1.40	1.44	3.79	0.274		0.008	< 0.001	2.00B	Y = 3.7064 − 0.0995x + 0.0009x^2^
**Proportion of lactic acid:acetic acid (LA:AA)**											
BRS	2.53	11.08	9.98	6.25	1.19	0.906		< 0.001	< 0.001	6.21B	Y = 0.4401 + 0.3712x − 0.0034x^2^
C70	0.01	11.53	10.11	14.10	1.19	1.311		< 0.001	< 0.001	7.39A	Y = 2.1684 + 0.4769x − 0.005x^2^
C60	0.11	11.48	10.71	2.48	1.19	1.254		0.148	< 0.001	5.19C	Y = 0.8567 + 0.4019x − 0.0037x^2^
C50	0.39	16.30	10.20	5.87	1.19	1.416		0.015	< 0.001	6.79AB	Y = 0.6049 + 0.4856x –0.0045x^2^
**Propionic acid (PA)**											
BRS	0.66	0.13	0.00	0.20	0.23	0.049		< 0.001	< 0.001	0.24C	Y = 0.2888 − 0.0133x + 0.0002x^2^
C70	1.32	0.16	0.15	0.25	0.23	0.104		< 0.001	< 0.001	0.42A	Y = 0.3417– 0.0187x + 0.0003x^2^
C60	0.31	0.80	0.24	0.64	0.23	0.054		< 0.001	< 0.001	0.44A	Y = 0.2568 + 0.0108x + 0.00004x^2^
C50	0.58	0.16	0.29	0.19	0.23	0.035		< 0.001	< 0.001	0.29B	Y = 0.2514 − 0.0051x + 0.00005x^2^
**Butyric acid (BA)**											
BRS	1.54	0.11	0.05	0.08	0.06	0.134		< 0.001	< 0.001	0.37B	Y = 0.1884 − 0.0214x + 0.0003x^2^
C70	5.43	0.08	0.14	0.10	0.06	0.491		< 0.001	< 0.001	1.16A	Y = 0.5256– 0.0777x + 0.0012x^2^
C60	0.74	0.25	0.48	0.31	0.06	0.053		< 0.001	0.271	0.37B	Y = 0.1112 -0.0052x
C50	0.10	0.06	0.28	0.06	0.06	0.020		< 0.001	< 0.001	0.11C	Y = 0.0467 + 0.0045x − 0.00005x^2^
**Ethanol**											
BRS	0.28	0.68	1.25	3.01	1.76	0.220		< 0.001	< 0.001	1.40C	Y = -0.0002x^2^ + 0.003x + 2.1546
C70	2.18	2.75	1.14	4.91	1.76	0.302		0.014	0.002	2.55A	Y = -0.0002x^2^ + 0.0183x + 2.5192
C60	0.64	0.54	0.76	0.96	1.76	0.104		< 0.001	< 0.001	0.93D	Y = 0.0002x^2^– 0.031x + 1.7216
C50	2.68	0.64	0.77	1.71	1.76	0.174		0.006	< 0.001	1.51C	Y = 0.0006x^2^– 0.054x + 2.0744
**N-NH**_**3**_**/NT**											
BRS	10.25	4.03	2.55	4.48	0.30	1.015	< 0.001		< 0.001	4.32A	Y = 1.5013– 0.0077x + 0.0009x^2^
C70	10.84	1.12	1.65	4.73	0.30	1.202	< 0.001		< 0.001	3.73AB	Y = 2.1096– 0.0801x + 0.0015x^2^
C60	9.36	2.38	1.57	4.09	0.30	1.038	< 0.001		< 0.001	3.54B	Y = 1.6438– 0.0453x + 0.0011x^2^
C50	10.65	2.16	1.57	1.15	0.30	1.005	< 0.001		< 0.001	3.16B	Y = 1.0298– 0.0897x + 0.0018x^2^

*SEM* standard error of the mean; means followed by different uppercase letters in the same column and lowercase letters in the same row differ statistically at the 5% probability level.

The acetic acid content in silage were maximum in the silage with 100% sugarcane, except for silage of the genotype C60 that showed a concentration of 4.65% in 75% of sugarcane inclusion. The BRS soybean genotype presented a positive quadratic effect (P < 0.001), however the silage with C70, C60, and C50 soybean genotypes, showed a negative quadratic effect (P ≤ 0.047). The average of acetic acid content in the silage with the C60 soybean genotype was higher than the other genotypes (P < 0.05).

The proportion of lactic and acetic acids, in all silages with soybean genotypes, exhibited a positive quadratic effect (P < 0.001), with maximum points of 11.08, 14.10, 11.48 and 16.30 at the inclusion level of 25% to BRS, C60, and C50 soybean genotypes, and inclusion level of 50% of sugarcane to C70 soybean genotype (Table 2). Also, silage with the C70 soybean genotype showed a higher (P < 0.05) average proportion of LA:AA.

Regarding propionic acid, the inclusion of sugarcane indicated a negative quadratic effect (P < 0.001) in all silages with soybean genotypes, with minimum points of 0.00, 0.15, 0.23, and 0.19% at the inclusion levels 25% of sugarcane to BRS and C70 soybean genotypes, 100% of sugarcane inclusion to C60, and 75% of sugarcane inclusion to C50 genotype. Silages with C70 and C60 soybean genotypes exhibited greater average of propionic acid (P < 0.05) (Table 2).

The production of butyric acid presented a negative quadratic effect (P < 0.05) on silage with BRS 333, C70, and C50 soybean genotype with minimum points of 0.05, 0.06, and 0.06% at the inclusion level 25% and 100% to BRS 333, C70 and C50 soybean genotypes, respectively. However, silage with the C60 soybean genotype showed a decreasing linear effect (P < 0.001) at the inclusion of sugarcane (Table 2). Silage with C70 soybean genotype indicated the highest average of butyric acid (P < 0.05).

The inclusion of sugarcane in silages with BRS and C70 soybean genotypes showed a positive quadratic effect for ethanol levels (P ≤ 0.002) with maximum points of 3.01 and 4.91 at the inclusion of 75% to BRS 333 and C70 soybean genotypes (Table 2). While silages with C60 and C50 soybean genotypes presented a negative quadratic effect (P < 0.001), with minimum points of 0.54 and 0.64% with the inclusion of 25% of sugarcane and silage. The C70 soybean genotype had the highest ethanol means (P < 0.05) (Table 2).

All silages with soybean genotypes showed a negative quadratic effect (P < 0.001) for the ammoniacal nitrogen (N-NH_3_/NT) levels of the silage. Silages with the BRS and C70 soybean genotypes exhibited the highest levels of ammoniacal nitrogen. Besides, silage with the BRS soybean genotype showed a minimum point of 2.55%; with 50% of sugarcane inclusion, and silage with the C70 soybean genotype presented a minimum point of 1.12%; with the inclusion of 50% of sugarcane. While silages of genotypes C50, C60, and C70 recorded lower (P < 0.001) values for N-NH_3_/NT.

The highest values of ammoniacal nitrogen were noticed for silage with 0% of sugarcane inclusion (10.27%). On the other hand, the lowest values were found for silages with 100% sugarcane (0.30%). However, for the other inclusion levels of sugarcane (25%, 50%, and 75%), the values remained below 5% of dry matter in silages (Table 2).

Furthermore, the interaction effect was observed between addition levels of sugarcane and soybean genotypes for pH values and gas losses (Table 3). There were no effects of inclusion levels of sugarcane (P = 0.542) and genotypes (P = 0.058) for effluent losses in silages as well as there was no interaction (P = 0.422) between the soybean genotypes and the levels of sugarcane in the silage.

**Table 3:** Probability values for Sugarcane levels, soybean genotypes, and the interaction between them.

^1^VS	pH	^2^GL	^3^EL	^4^DMR
Levels	< 0.001	0.271	0.542	< 0.001
Genotypes	0.732	< 0.001	0.058	0.543
N^5^ × G^6^	0.002	< 0.001	0.422	0.133

^1^Variation source; ^2^gas losses; ^3^effluent losses; ^4^dry matter recovery, ^5^sugarcane levels; ^6^soybean genotypes.

The inclusion of sugarcane promoted a negative quadratic effect (P < 0.001) for the pH value of C70 soybean genotype, with a minimum point of 3.40 in the inclusion of 0% of the sugarcane added. For the other genotypes, a linear decreasing effect was observed (P < 0.001) (Table 4).

**Table 4:** Mean values of pH, gas losses (GL), and dry matter recovery (DMR) of silages of soybean genotypes with levels of added sugarcane.

Soybean genotypes	Inclusion levels in sugarcane					SEM	P value		Mean	Equation
	0	25	50	75	100		L	Q		
**pH**										
BRS333	5.32	3.94	3.75	3.56	3.40	0.164	< 0.001	< 0.001	3.99A	Y = 3.1465 –0.0169x
C70	5.66	3.55	3.74	3.86	3.40	0.190	< 0.001	< 0.001	4.04A	Y = 3.6569 − 0.02x + 0.0004x^2^
C60	5.57	3.93	3.63	3.58	3.40	0.185	< 0.001	< 0.001	4.02A	Y = 3.079 − 0.0188x
C50	5.23	3.88	3.75	3.74	3.40	0.148	< 0.001	< 0.001	4.00A	Y = 3.236 − 0.0153x
**Gas losses (dag/kg)**										
BRS333	6.94	6.44	9.83	15.25	9.93	0.847	< 0.001	0.091	9.68A	Y = 6.72 + 0.0592x
C70	7.69	6.10	6.81	14.32	9.93	0.894	0.015	0.828	8.97A	Y = 11.508 − 0.0508x
C60	7.63	9.86	9.49	13.33	9.93	0.720	0.102	0.218	10.05A	Y = 10.05
C50	14.18	2.27	8.70	9.59	9.93	1.064	0.807	0.004	8.93A	Y = 11.404 − 0.2118x + 0.0022x^2^
**Dry matter recovery (%)**										
BRS333	93.61	94.49	95.71	84.74	83.28	2.502	< 0.001	0.020	90.37A	Y = 81.8831 + 0.3140x − 0.0019x^2^
C70	89.58	90.86	94.63	89.18	83.28	3.004	0.019	0.001	89.51A	Y = 83.2496 + 0.3266x − 0.0026x^2^
C60	90.22	93.14	95.43	93.03	83.28	1.913	0.022	< 0.001	91.02A	Y = 83.9410 + 0.3991x − 0.0034x^2^
C50	85.85	93.72	95.39	89.71	83.28	3.372	0.129	< 0.001	89.59A	Y = 82.6326 + 0.4471x − 0.0041x^2^

*SEM* standard error of the mean; means followed by different uppercase letters in the same column and lowercase letters in the same row differ statistically at the 5% probability level.

About the gas losses, the silages with C60 soybean genotype did not exhibit statistical effects (P = 0.102) with the addition of sugarcane. While silages with BRS 333 and C70 genotypes showed an increasing linear effect (P ≤ 0.015). Silage with the C50 soybean genotype showed a negative quadratic effect with a minimum point of 2.27 dag/kg DM for gas losses, at the sugarcane inclusion level of 25% (P = 0.004) (Table 4).

The dry matter recovery (DMR) presented a positive quadratic effect (P ≤ 0.020) for the sugarcane addition in silages with soybean genotypes. The addition of 50 and 25% of sugarcane in the soybean silage indicated higher values of DMR, ranging from 93.05 to 95.29%, respectively (Table 4).

Also, there was an interaction between sugarcane levels and soybean genotypes for levels of dry matter (DM), mineral matter (MM), and crude protein (CP) (P < 0.001) (Table 5). The levels of neutral detergent fiber (NDF) were influenced by genotypes (P = 0.017) and levels of sugarcane (P = 0.010) were no influenced by interaction between levels of sugarcane and soybean genotypes (P = 0.089). The acid detergent fiber (ADF) were affected only by the genotypes (P = 0.020) (Table 5).

**Table 5:** Probability values for levels of sugarcane, soybean genotypes, and of interaction for the chemical composition of silages.

^1^VS	^2^DM	^3^MM	^4^CP	^5^NDF	^6^ADF	^7^WSC	^8^IVDMD
Levels	< 0.001	0.001	< 0.001	0.010	0.080	< 0.001	< 0.001
Genotypes	0.002	< 0.001	< 0.001	0.017	0.020	< 0.001	< 0.001
^9^ N × ^10^G	< 0.001	< 0.001	< 0.001	0.089	0.902	< 0.001	< 0.001

^1^Variation source; ^2^Dry matter; ^3^Mineral matter; ^4^Crude protein; ^5^Neutral detergent fiber; ^6^Acid detergent fiber; ^7^Water-soluble carbohydrates; ^8^In vitro of DM digestibility; ^9^Sugarcane levels; ^10^Soybean genotypes.

Silages with BRS 333 and C50 soybean genotypes, showed a positive quadratic effect (P ≤ 0.020) for the DM content (Table 6), with a maximum value of 20.40 and 19.93%, at the sugarcane inclusion levels of 25%. While, silage with the genotype C60 did not present statistical differences for the DM contents with the inclusion of sugarcane (P = 0.379). However, silage with the C70 genotype exhibited a decreasing linear effect with the inclusion of sugarcane (P = 0.035).

**Table 6:** Mean values of the chemical composition of soybean genotype silages with levels of added sugarcane.

Soybean genotypes	Inclusion levels in sugarcane					SEM	P value		Mean	Equation
	0	25	50	75	100		L	Q		
**Dry matter (DM) %NM**										
BRS	19.30	20.40	19.28	18.03	15.99	0.434	0.001	0.020	18.60A	Y = 15.887 + 0.1092x − 0.0007x^2^
C70	15.06	13.14	18.39	16.81	15.99	0.501	0.035	0.120	15.88B	Y = 16.986 − 0.0221x
C60	16.04	16.22	15.05	15.57	15.99	0.273	0.717	0.379	15.78B	Y = 15.78
C50	15.65	19.93	19.05	17.64	15.99	0.478	0.489	< 0.001	17.65A	Y = 15.562 + 0.1478x − 0.0014x^2^
**Mineral matter (MM) %DM**										
BRS	8.72	7.6	7.27	6.8	5.84	0.229	< 0.001	0.808	7.25C	Y = 5.9332 − 0.0263x
C70	9.08	7.19	7.67	10	5.84	0.357	0.005	0.056	7.96A	Y = 6.2202 − 0.0175x
C60	10.48	9.09	7.71	6.04	5.84	0.425	< 0.001	0.081	7.83AB	Y = 5.3633 − 0.0493x
C50	9.98	7.55	7.07	8.12	5.84	0.342	< 0.001	0.138	7.71B	Y = 5.3572 − 0.039x
**Crude protein (CP) %DM**										
BRS	7.09	6.20	4.81	3.37	1.35	0.471	< 0.001	< 0.001	4.56C	Y = 1.7016 + 0.0572x
C70	6.53	4.79	5.86	6.85	1.35	0.482	< 0.001	< 0.001	5.07B	Y = 2.4981 + 0.1018x − 0.0007x^2^
C60	6.64	6.76	5.68	3.82	1.35	0.481	< 0.001	< 0.001	4.85BC	Y = 1.2916 + 0.1223x − 0.0007x^2^
C50	10.54	12.17	9.41	4.36	1.35	0.942	< 0.001	< 0.001	7.57A	Y = 0.6725 + 0.2371x − 0.0013x^2^
**Neutral detergent fiber (NDF) %DM**										
BRS	45.78	48.83	49.00	53.28	56.82	1.103	< 0.001	0.422	49.22B	Y = 45.436 + 0.1061x
C70	49.93	56.50	50.86	51.04	56.82	1.054	0.211	0.581	52.08A	Y = 53.03
C60	42.21	48.93	47.16	51.12	56.82	1.423	< 0.001	0.669	47.35B	Y = 42.966 + 0.1256x
C50	50.05	47.86	48.82	49.09	56.82	0.924	0.004	0.002	48.96B	Y = 50.310 − 0.1598x + 0.0022x^2^
**Water-soluble carbohydrates (WSC) %DM**										
BRS	4.22	6.72	11.47	13.97	20.54	0.233	< 0.001	0.808	11.38B	Y = 0.243 + 3.989x
C70	4.81	7.31	12.55	15.05	20.54	0.398	< 0.001	0.556	12.04A	Y = 0.619 + 3.923x
C60	3.78	6.28	11.28	13.78	2054	0.344	< 0.001	0.581	11.13B	Y = 0.834 + 4.102x
C50	5.01	7.51	12.51	15.01	20.54	0.367	< 0.001	0.128	12.11A	Y = 0.888 + 3.856x
**In vitro of DM digestibility (IVDMD) %DM**										
BRS	61.78	62.55	63.52	61.07	59.97	1.15	< 0.089	< 0.001	61.77B	Y = 59.706 + 3.1303x − 0.6337x^2^
C70	63.56	64.33	65.3	62.85	59.97	1.09	< 0.065	< 0.001	63.20A	Y = 61.486 + 3.1305x − 0.6253x^2^
C60	62.58	63.35	64.32	61.87	59.97	1.13	< 0.087	< 0.001	62.41B	Y = 60.506 + 3.1255x − 0.6299x^2^
C50	64.48	65.25	66.22	63.77	59.97	0.98	< 0.001	< 0.001	63.93A	Y = 61.332 + 4.3066x − 0.8091x^2^

*SEM* standard error of the mean; means followed by different uppercase letters in the same column and lowercase letters in the same row differ statistically at the 5% probability level.

It was possible to note that all silages with soybean genotypes had a decreasing linear effect (P < 0.01) with the inclusion of sugarcane for MM levels, with the highest (P < 0.001) MM levels observed in silages of the C70 genotype (7.96%) (Table 6).

A decreasing linear effect (P < 0.001) of the CP contents with the inclusion of sugarcane in the silage with BRS 333 soybean genotype was observed. Moreover, for silages with C70, C60, and C50 soybean genotypes, a positive quadratic effect (P < 0.001) was noticed, with maximum points of 6.85, 6.76, and 12.17% in the levels of sugarcane inclusion of 75, 25 and 25%, respectively. The silages with the C50 soybean genotype exhibited a higher mean (P < 0.001) for the CP contents compared to the other cultivars, 7.57% (Table 6).

Silage with 100% sugarcane presented the highest mean (P < 0.05) for the NDF contents (56.82%). Still, a lower mean (P < 0.05) was observed in the NDF contents for the BRS 333, C60, and C50 genotypes. The BRS 333 soybean genotype had the lowest mean (P < 0.05) for ADF levels (34.90%) compared to the others soybean genotype.

An increasing linear effect (P < 0.001) was observed for values of WSC with the inclusion of sugarcane in silage for all soybean genotypes (Table 6).

There was a quadratic effect (P < 0.001) for the IVDMD values with the inclusion of sugarcane in silage for all soybean genotypes. The silages with C70 and C50 soybean genotype showed a higher mean (P < 0.05) for IVDMD values, than silages with C60 and BRS333 soybean genotypes (Table 6).

## Discussion

### Effect of addition of sugarcane

The addition of sugarcane reduced the pH of the silages, probably due to the increase in levels of WSC, which sugarcane has a high content. According to^13^, the values of WSC in sugarcane are high, so the sugars contribution enables an accelerated fermentation in ensiled biomass, providing rapid proliferation of lactic acid bacteria under anaerobic conditions.

The rapid proliferation of lactic acid bacteria, mainly homofermentative bacteria, influences the enhance of the lactic acid concentration, causing a considerable reduction in the pH value^17,18^.

In silages with 100% sugarcane, the pH value presented, on average, 3.40; that is, below that recommended by^18^. ^19^explain that silages with values below 3.80 tend to have a predominance of fungi, mainly yeasts, which can ferment sugar to ethanol, reducing the nutritional value of silage, besides increasing losses by gases, and effluents. Thus, it is noted that the silages with higher proportions of sugarcane had lower pH and higher levels of ethanol (Table 2) and WSC (Table 6). ^16^reported that the smaller production of ethanol may be inhibited by the acetic acid produced during fermentation. Sugarcane when ensiled at different stages of maturation promotes less recovery of dry matter than when ensiled together with other forages or with additives^14,16,19–21^, corroborating the results in Table 4.

^16^reported that the sugarcane is preferably harvested at the stage of maturity above 18 months and during the dry period to have a greater amount of DM and WSC associated with levels below 2% of CP, which could cause a silage with high ethanol concentration. ^22^reported that the sugarcane harvest time is related to the concentration of WSC, when harvested in the rainy season there is a reduction in the concentration of WSC. ^16,18,20,22^claimed that enterobacteria predominant in the initial stage of forage fermentation can cause proteolysis, which also hinder the pH drop even with the LAB acting on LA and AA, slowing the pH slower and thus reducing the substrate for the yeast proliferation and alcoholic fermentation, it can be inferred that the silage with 100% sugarcane also presented higher AA values ​​that also inhibit the presence of yeasts, thus causing lower ethanol values, and corroborate the results reported by^23^.

Nonetheless, silages produced exclusively with soybean genotypes exhibited higher pH values due to the chemical characteristics of the soybean plant. According to^7,9,11,12^, legumes with an elevated concentration of crude protein, for instance, soybeans, have a high buffering capacity. This characteristic makes it difficult to reduce the pH value leading to butyric fermentation and ammoniacal nitrogen production, which is not desired in the fermentation process of silage since it stimulates dry matter, fermentative, and nutritional losses. Silages added with 0% of sugarcane, in the present study, showed higher values for pH, CP, butyric acid, and N-NH_3_/NT, corroborating the statement of the authors above mentioned. Thus, the possibility of proliferation of undesirable bacteria, such as clostridia and enterobacteria, increased in silages containing 0% of sugarcane, which promotes secondary fermentation, mainly proteolysis, which generates butyric acid and N-NH_3_/NT as a by-product^9^.

The combination of sugarcane and soybean provided an environment conducive to the development of LAB causing a high concentration of LA, however the joint production of AA, that inhibited the presence of yeasts and thus controlled the production of ethanol. The high LA production in soybean silage with sugarcane levels ranging in 25–75%, contradicting the tendency of classical fermentation to promote sharp pH drop, associated to lower pH values was not observed possibly due to the presence of a synergism between these parameters that modified the pH drop, which was modulated by the buffering power of soybean silage, as legumes in general^7,22^. The large amount of proteins acts neutralizing the hydrogen ions from the organic acids, in particular LA produced in the silage fermentation, modulating the pH drop and causing greater transformation of WSC into lactic acid, without a sharp decreased in the pH, also decreasing the availability of WSC to be fermented into ethanol (Table 4), that corroborate the findings reported by^7,16,22,23^.

^12^evaluating the fermentation profile of mixed corn and soybean silages observed that the pH value varied between 3.69 and 4.51, demonstrating that silages with a higher proportion of soybeans favored the increase in the pH of the silage.

All levels of sugarcane inclusion showed a reduction in ammonia production, with levels less than 10% of N-NH_3_/NT; according to^18^, it is acceptable in silages as it does not cause intoxication and reduces voluntary consumption of animals. Nevertheless, the inclusion of sugarcane, at the level of 50% of soybean plants, resulted in the lower production of N-NH_3_/NT.

Providing an environment to stimulate the rapid growth of lactic acid microorganisms is crucial to decrease nutritional losses and increase silage management efficiency^17,18^. Thus, the inclusion of sugarcane in soybean silage promoted an adequate supply of soluble carbohydrates (Table 6), which are substrates for lactic acid bacteria to produce lactic acid. In the present study, it was possible to observe that the inclusion of 50% of sugarcane favored the recovery of dry matter around 95% for soybean silages, evidenced by the higher production of lactic acid for this level (15.53%). However, silages with sugarcane proportions above 75% exhibited a reduction in RMS, which was probably due to higher concentrations of soluble carbohydrates, leading to alcoholic fermentation, which is undesirable. Consequently, higher values for ethanol and gas losses were observed for the inclusion of 75 and 100% of sugarcane in soybean silage.

Gas losses are due to the fermentation of carbohydrates and proteins, leading to the metabolic production of CO_2_, N_2_O and N-NH_3_, representing most of the total DM losses, which may exceed 90% of the total^19^. Several studies^19–23,25,26^ indicated that gas losses are generally close to 10% of dry matter, being influenced by the fermentative profile of ensiled material. The lower gas losses occur when desirable microorganisms, such as homofermentative bacteria, are predominant. It makes difficult the development of gas-producing microorganisms, as yeast; also, these gas losses are considered minimal^18,25^. In the present study, there were no significant differences (P > 0.05) for gas losses between the averages of the inclusion levels; however, higher values were observed for silage with 75% of sugarcane (13.12 dag/kg).

The addition of sugarcane in any proportion did not change the effluent losses (EL) in soybean silages. This fact can be explained due to the similarity of the dry matter content of sugarcane with the soybean genotypes in pre-silage, varying between 16.32 and 18.00% DM.

Therefore, there was no influence in silage moisture enough to interfere with the effluent losses from the silage. The high forage moisture can favor the development of harmful microorganisms, such as clostridia, affecting the fermentative stability inside the silo, which, besides losses by effluents, results in lower silages quality^27^.

According to^18^, the ensiled mass has to contain dry matter contents between 28 and 35% to guarantee an adequate lactic fermentation, fewer losses by effluents and nutrients due to the inhibition of undesirable microorganisms, such as clostridia, which produce butyric acid. Silage with 100% soybean presented an average (1.95%) above the recommended range as ideal for butyric acid, which is less than or equal to 1.0%, according to^28^. Nevertheless, the explanation for the higher levels of butyric acid in silage with 100% soybean could be related to the high pH value observed for this silage, which, probably, implies in the greater clostridia activity, that operates in pH ranges superior to 5.0. It was observed that the inclusion of 25% of sugarcane was sufficient to reduce the levels of pH and butyric acid in the silages, indicating a possible inhibition of clostridia and leading to fermentation for lactic acid, due to the supply of WSC (Table 6).

The high propionic acid concentrations (0.72%), were observed in silage with 100% of soybean. According to^17^, it can be classified as high since it is above 0.3%; thus, it can act as indicative of silages with secondary fermentation, due to the possible action of harmful microorganisms, commonly observed in silages with a predominance of clostridial fermentation.

About chemical characteristics, as the proportion of soybean increased in silage, the concentration of CP, MM, and IVDMD also increased to medium levels of sugarcane inclusion. The results obtained in the present study are in accordance with those found by^12^, in which soybean increased the crude protein content in mixed silage with millet.

Thus, the inclusion of sugarcane had a negative influence on the nutritional value of silage at higher levels of sugarcane, due to the characteristics of the plants in the pre-ensiling, in which sugarcane presented lower levels of crude protein and mineral material compared to soybean, but within the appropriate inclusion level, it was effective in fermentation balance.

The harvesting of sugarcane in the rainy season at a time similar to the soybean plant promoted improvements in the fermentation profile of soybean silage by adding levels of sugarcane inclusion due to the addition of sufficient WSC content to promote synergism between microorganisms^16^, promoting classical silage fermentation characterized by an increase in the concentration of lactic acid and a pH close to that recommended by^18^, 4.2 and 3.8, minimizing the occurrence of undesirable fermentations.

### Comparison among genotypes

Silages of all soybean genotypes in isolation exhibited a high DMR (mean of 90.12%). However, it is noteworthy that, the inclusion of sugarcane provided an additional and complementary effect for the DMR of the silage of the two mixed forages since the DMR of sugarcane silage had a lower value (83.28%). Other studies confirm that these same forages ensiled in isolation had lower DMR, for instance, in a study conducted by^28^, who evaluated the preservation of the sugarcane control silage, observed a DMR of 78.40%. Moreover, the observations of^3^, studying the fermentative profile of soybean silages, found DMR of 91.52% for soybean silage, values close to the present study.

Therefore, the mixed silage resulted in lower losses during silage, corroborating the hypothesis of this study, in which the factors that hinder the silage process of the isolated soybean and sugarcane plant are canceled with the appropriate combination in the silage (Table 2). According to^12^, a combination of 60% of millet and 40% of soybean improves the crude protein content and the fermentability of silages.

Silages with the BRS and C50 soybean genotypes showed higher mean for DM contents, though, the silage values are below the recommended by^18^, indicating that the inclusion of sugarcane was not effective in reducing the moisture content of ensiled biomass. The silage of genotype C50 exhibited superiority for the levels of CP and IVDMD, thus, indicating it cultivar had the greatest capacity to preserve its nutritional value. However, this is not due to genotypic aspects, because, the soybean genotypes showed low variation in CP levels in the pre-silage moment; therefore, the superiority of CP in silage of C50 genotype is due to the silage fermentation issues. Besides, the C50 genotype showed a lower numerical value for N-NH_3_/NT, which indicates that there was less proteolysis in the silage and lower values for NDF and ADF.

Assessing the chemical composition of soybean and millet silages^18^, observed values ranging from 14.0 to 16.5% of crude protein, with the maximum values for silage with 100% soybean. The present study presented lower average values for soybean silage (7.70% of CP), although, in the pre-silage, the crude protein content was 15.29%. The reduction of CP content in silage, in this study, is due to the low DM content of the soybean plant at the time of pre-ensiling, 16.86%, compared to the research of^18^, which observed a DM content of 23.10% for soybean silage.

The silage of the C70 genotype showed a higher production of lactic acid, propionic acid, butyric acid, and ethanol, except for acetic acid. This fact may be linked to a higher presence of WSC (Table 6) for this cultivar, increasing the supply of substrates for the microorganisms to act in the silage fermentation^29^. However, the higher production of butyric acid above 1% may be related to the low dry matter content (15.88%) compared with the other silages. According to^27^, butyric acid should not be observed in silage that has been properly fermented, as it indicates the activity of clostridia, which in addition to increasing dry matter losses, may cause proteolysis.

## Conclusion

The highest values of dry matter recovery, fermentative profile, and levels of crude protein and digestibility occurred in the inclusion between 25 and 50% of sugarcane in soybean silages.

The C50 soybean genotype stood out to exhibit superiority concerning other genotypes and also to present higher values of biomass production.

## Methods

The experiment was carried out at the Center for Agricultural and Environmental Sciences, at the Federal University of Maranhão, in the municipality of Chapadinha, Brazil. According to Köppen's classification, the climate is Aw’, characterized by a tropical rainy climate with dry-winter and rainy-summer. Soybean genotypes and sugarcane were cultivated in 2017. The Soybean genotypes were from commercial areas in partnership with the Federal University of Maranhão, located in the municipality of Chapadinha, the eastern region of the state of Maranhão (3.6° 86′ S and 43° 14′ O). The experimental trials were conducted between 2017 and 2018. The sugarcane was grown in the Experimental area of agrarian sciences center (Federal University of Maranhão, Chapadinha-Maranhão).

The experiments were conducted in a completely randomized design (CRD), in factorial scheme 4 × 5 (four soybean genotypes and five levels of sugarcane inclusion), with four replications, totaling 80 experimental plots (silos). The soybean genotypes used were Pampeanas C50, C60 and C70, and the cultivar BRS 333. Moreover, sugarcane was included in the cultivars of whole soybean plant at levels of 0, 25, 50, 75, and 100%, based on natural matter (NT). The agronomic characteristics of soybean crops used in silage are shown in Table 7.

**Table 7:** Agronomic characteristics of soybean genotypes used in the experimental trial.

	NMY^1^	DM^2^	DMY^3^	Pods (no./sampling)^4^	Proportion of plant components (DM)		
					%S^5^	%L^6^	%P^7^
BRS333	30,800.00	17.35	5343.8	363	44.30	30.63	25.06
C70	14,210.00	15.69	2229.6	14	51.47	46.64	1.87
C60	13,220.00	16.32	2157.5	0	51.87	48.12	0
C50	27,000.00	18.08	4881.6	180	54.13	28.20	17.66

^1^Natural matter yield per hectare (kg/ha); ^2^dry matter percentage; ^3^dry matter yields; ^4^pods (no./sampling) are number of pods counted in a sampling area of one square meter ; ^5^percentage of stem; ^6^percentage of leaves; ^7^percentage of pods.

Soybean genotypes were sown mechanically, in January 2017, with an inter-row spacing of 0.50 m. The seeder was adjusted to 13 plants per linear meter, totaling a stand of 260,000.00 plants per hectare. The rainfall recorded in the site was 640 mm for the period between planting and harvesting. At 75th days after planting (DAP), the soybean genotypes were harvested by cutting plants at a height of 10 cm above the soil surface, in the R-5:3 stage of phenological development, according to the recommendation of^7,30^. Sugarcane plants were planted with a spacing of 0.8 m inter-rows and harvested at 18 months after planting.

Soybean and sugarcane plants were chopped using a stationary chopper, with particle sizes of approximately 2 cm and, posteriorly, weighed and incorporated according to the level of inclusion proposed for experimental treatments. The chopped material was homogenized and was mixed by hand.

Silage was made using experimental silos of polypropylene buckets with a capacity of 3.5 kg. At the bottom of the mini-silo, 1 kg of sand was placed to capture the effluents, separated from the forage by cotton fabric. The mini-silos were sealed with a lid equipped with a Bunsen valve, ensuring hermetic sealing and elimination of gases generated inside the mini-silos during the silage fermentation process. The silos were stored for 70 days in a Lab room at Federal University of Maranhão, with controlled temperature (25 ± 2 °C). Losses of DM in silages in the form of gases and effluents and the DMR were estimated according to^24^. DMR was calculated through equations:

$$DMR=\frac{ (FMo \times DMo)}{(FMs \times DMs)} \times 100$$

which DMR = dry matter recovery rate (%); FMs = forage mass at sealing (kg); DMs = forage dry matter concentration at sealing (%); FMo = forage mass at opening (kg); DMo = forage dry matter concentration at opening (%)^24^.

Samples of the material were collected at the time of silage and after silo opening periods, and then, dried in a forced-ventilation oven at a temperature of 65 °C for 72 h. The samples were individually processed with a knife mill at 1 mm mesh sieve to determine the chemical composition. The analyzes were carried out at the Animal Products Laboratory (LAPOA) of the Center for Agricultural and Environmental Sciences at the Federal University of Maranhão.

According to the specific protocols for each parameter, the following analyses were made: DM, using an oven at 105 °C, by method No. 934.01^31^; MM, by method No. 930.05^31^; and CP, by the method of Kjeldahl, n ° 981.10^31^.

The components of the cell wall, NDF and ADF were analyzed in conformity with the methods of^32,33^. Hemicellulose levels were determined by the differences between NDF and ADF. Chemical composition of sugarcane and soybean genotypes before ensiling are shown in Table 8.

**Table 8:** Chemical composition of sugarcane and soybean cultivars at the time of silage.

%DM	Soybean genotypes				Sugarcane
	BRS 333 RR	C70 RR	C60 RR	C50 RR	
DM (%NM)	17.35	15.69	16.32	18.08	18.00
OM	91.70	91.33	86.00	90.33	96.80
MM	8.30	8.67	14.00	9.67	3.20
CP	15.09	14.45	16.04	15.58	2.47
NDF	51.79	59.79	54.38	48.44	45.36
ADF	48.60	49.60	41.74	45.26	34.13
Hemicellulose	3.19	10.19	12.64	3.18	11.23

%*DM* percentage on the dry matter, *NM* based on the natural matter, *OM* organic matter, *MM* mineral matter, *CP* crude protein, *NDF* neutral detergent fiber, *ADF* acid detergent fiber.

A fresh silage sample was used to evaluate pH and ammoniacal nitrogen. To pH analyses, 25 g were collected on the central part of the silos. After that, the sample was homogenized in 75 mL of distilled water, where it was kept for 30 min. The reading was done using a potentiometer^34^.

The N-NH_3_ determination was carried out according to the methodology of^35^, in which a fraction of 25 g of silage sample was mixed with 100 mL of 2 mol/L potassium chloride solution for 10 min, then, filtered and 10 mL were collected. This material was transferred to a digester tube containing 250 mg of calcined magnesium oxide, later, distilled to capture the ammonia and then titrated to quantify the N-NH_3_/NT in percentage.

Organic acids (acetic, propionic and butyric), lactic acid and ethanol were determined using the methodology described by^36^, using high-performance liquid chromatography (HPLC, Shimadzu Scientific Instruments, Columbia, MD), detector model SPD-10A VP coupled to a detector (UV), using shortest wavelength (210 nm). Water-soluble carbohydrates (WSC) were determined according to the methodology of^37^.

IVDMD was determined according to^38^ by weighing 0.5 g of pre-dried samples in previously dried and calibrated centrifuge tubes. To the tubes, 40 mL of McDougall's solution (artificial saliva) and 10 mL of rumen inoculum of animals kept in signal grass pastures (*Brachiaria decumbens* cv.) with mineral salt in the trough were added.

The tubes were closed with rubber stoppers containing a Bunsen valve (immediately after passing CO_2_) and incubated for 48 h in a controlled temperature oven, where they were shaken at least four times during fermentation. The second phase occurred after centrifuging and discarding the supernatant. A solution of pepsin (1:10,000) was added (50 mL) at 0.2% to each tube. Then, they have been shaken and placed in an oven at 39° C to 48 h. After washing, drying and weighing of the tubes, calculations were made according to the following equation:

$$IVDMD=\frac{ 100 \times g of DM at sample- (\mathrm{g of DM residual }-\mathrm{ g of DM white})}{g of DM at sample}$$

The effects of silage fermentation on losses data, organic acids, and chemical composition were assessed using the variance analysis procedures (ANOVA), and regression with orthogonal contrasts and, using the PROC GLM and PROC REG commands^39^ with 5% significance.
